# Immunolocalization and Changes of Hydroxyproline-Rich Glycoproteins During Symbiotic Germination of *Dendrobium officinale*

**DOI:** 10.3389/fpls.2018.00552

**Published:** 2018-04-25

**Authors:** Yuan-Yuan Li, Xiao-Mei Chen, Ying Zhang, Yu-Hsiu Cho, Ai-Rong Wang, Edward C. Yeung, Xu Zeng, Shun-Xing Guo, Yung-I Lee

**Affiliations:** ^1^Institute of Medicinal Plant Development, Chinese Academy of Medical Sciences and Peking Union Medical College, Beijing, China; ^2^Biology Department, National Museum of Natural Science, Taichung, Taiwan; ^3^Department of Biological Sciences, University of Calgary, Calgary, AB, Canada; ^4^Department of Life Sciences, National Chung Hsing University, Taichung, Taiwan

**Keywords:** *Dendrobium*, immunolocalization, hydroxyproline-rich glycoproteins, mycorrhiza, symbiotic germination

## Abstract

Hydroxyproline-rich glycoproteins (HRGPs) are abundant cell wall components involved in mycorrhizal symbiosis, but little is known about their function in orchid mycorrhizal association. To gain further insight into the role of HRGPs in orchid symbiosis, the location and function of HRGPs were investigated during symbiotic germination of *Dendrobium officinale*. The presence of JIM11 epitope in developing protocorms was determined using immunodot blots and immunohistochemical staining procedures. Real-time PCR was also employed to verify the expression patterns of genes coding for extensin-like genes selected from the transcriptomic database. The importance of HRGPs in symbiotic germination was further investigated using 3,4-dehydro-L-proline (3,4-DHP), an inhibitor of HRGP biosynthesis. In symbiotic cultures, immunodot blots of JIM11 signals were moderate in mature seeds, and the signals became stronger in swollen embryos. After germination, signal intensities decreased in developing protocorms. In contrast, in asymbiotic cultures, JIM11 signals were much lower as compared with those stages in symbiotic cultures. Immunofluorescence staining enabled the visualization of JIM11 epitope in mature embryo and protocorm cells. Positive signals were initially localized in the larger cells near the basal (suspensor) end of uninfected embryos, marking the future colonization site of fungal hyphae. After 1 week of inoculation, the basal end of embryos had been colonized, and a strong signal was detected mostly at the mid- and basal regions of the enlarging protocorm. As protocorm development progressed, the signal was concentrated in the colonized cells at the basal end. In colonized cells, signals were present in the walls and intracellularly associated with hyphae and the pelotons. The precise localization of JIM11 epitope is further examined by immunogold labeling. In the colonized cells, gold particles were found mainly in the cell wall and the interfacial matrix near the fungal cell wall. Four extensin-like genes were verified to be highly up-regulated in symbiotically germinated protocorms as compared to asymbiotically germinated ones. The 3,4-DHP treatment inhibited the accumulation of HRGPs and symbiotic seed germination. In these protocorms, fungal hyphae could be found throughout the protocorms. Our results indicate that HRGPs play an important role in symbiotic germination. They can serve as markers for fungal colonization, establishing a symbiotic compartment and constraining fungal colonization inside the basal cells of protocorms.

## Introduction

Plant cell walls are composed of polysaccharides and other polymers that provide the protoplasm with structural support and protection (Taiz and Zeiger, 2010). In addition to carbohydrate polymers, plant cell walls also contain structural proteins, e.g., hydroxyproline-rich glycoproteins (HRGPs), proline-rich proteins (PRPs), glycine-rich proteins and arabinogalactan-proteins (AGPs) that modify the physical and chemical characteristics of cell wall in response to various developmental and environmental signals (Cassab, 1998). HRGPs occur in plant cell walls as a major protein component (Berger et al., 1994; Cassab, 1998). Extensins are cell wall proteins belonging to the superfamily of HRGPs, and they are known to participate in many processes during plant growth and development, such as pollen recognition and fertilization (Wu et al., 2001), cell division and differentiation (Ruiz-Avila et al., 1992), cell adhesion (Cassab, 1998), cessation of the cell growth (Cassab and Varner, 1987), and zygotic and somatic embryo development (Ruiz-Avila et al., 1991; Xu et al., 2011). Furthermore, HRGPs are involved in the interactions between plants and microorganisms, such as resistance to pathogens (Xie et al., 2011) and symbiotic associations (Balestrini et al., 1997). In the legume-rhizobium symbiosis, HRGPs are found to accumulate mainly in the walls of infected cells and in peribacteroid membranes surrounding groups of bacteroides (Benhamou et al., 1991; Rae et al., 1992), suggesting a crucial role in nodule development.

Endomycorrhizas, e.g., arbuscular mycorrhizas, ericoid mycorrhizas and orchid mycorrhizas, are characterized by intracellular hyphae growth and demonstrate a great diversity of infection patterns (Gianinazzi-Pearson, 1996; Peterson and Massicotte, 2004). In arbuscular mycorrhizas, the intracellular haustoria-like structures, known as arbuscules are formed by Glomeromycota, while in orchid mycorrhizas, intracellular hyphae coils, known as pelotons are produced by fungal species mostly belonging to Basidiomycetes, and these fungal hyphae are eventually digested by orchid cells (Smith and Read, 2008; Dearnaley and Cameron, 2017). During arbuscular mycorrhizal symbiosis, the arbuscules form an important symbiotic interface for nutrient exchange, and HRGPs have been located in the interfacial zone in addition to that in the peripheral walls of the host cells (Bonfante-Fasolo et al., 1991; Balestrini et al., 1994; Balestrini and Bonfante, 2005). These results suggest that HRGPs play a role in symbiotic associations, e.g., the formation of an interfacial compartment (Balestrini and Bonfante, 2014).

In the natural environment, germination of orchid seeds depends on appropriate mycorrhizal associations for nutrient supplies, e.g., carbohydrates and essential minerals (Smith and Read, 2008; Rasmussen and Rasmussen, 2009). Recent studies have revealed the occurrence of bidirectional nutrient transfer in orchid protocorms. Developing protocorm receives carbon and minerals nutrients, e.g., P and N from a mycorrhizal fungus (Cameron et al., 2006, 2007; Bougoure et al., 2013; Kuga et al., 2014) and returns NH_4_^+^ to its fungal partners (Fochi et al., 2017). In the mycorrhizal protocorm, nodulin-like genes were notably up-regulated, suggesting a role in carbon flow (Perotto et al., 2014). During symbiotic germination, nutrients are transported from the fungal hyphae to protocorm cells through the interfacial zone and the plasma membrane of orchid cells. There has been extensive research on the changes of nutrient reserves, organelles, plasma membrane, fungal structures and the organization of cytoskeleton in symbiotic germination of orchids (Smith, 1967; Peterson and Currah, 1990; Uetake et al., 1992; Peterson et al., 1998). Although cell wall remodeling is crucial during symbiotic interactions, until now, there is little information available on the distribution and the role of plant cell wall proteins during orchid symbiotic germination.

In this study, we investigate the distribution and the possible role of HRGPs on symbiotic germination of *Dendrobium officinale. D. officinale* has been used in the nobile-type dendrobium breeding and its dry stems are used in traditional Chinese medicine with a broad range of therapeutic effects (Pharmacopoeia Committee of People’s Republic of China, 2005). In our previous studies, we have developed an efficient symbiotic germination culture protocol of *D. officinale* to inoculate seeds with fungal strains (*Tulasnella* sp.) isolated by the *in situ* seed baiting technique (Wang et al., 2011; Tan et al., 2014). This procedure provides an ideal system for investigating the interactions between orchid seeds and their mycorrhizal fungal partners (Zhao et al., 2013; Chen et al., 2017). To gain a better insight into the possible functions of HRGPs in symbiotic germination of orchids, it is essential to determine their distribution and to study their biological function during symbiotic interaction. In this study, immunolocalization of JIM 11 epitope was performed to investigate the distribution of HRGPs during symbiotic germination of *D. officinale*. The JIM11 epitope, which recognizes specific arabinosylation motifs of HRGPs has been successfully used to localize extensins during embryo development of monocots, i.e., *Musa* (Xu et al., 2011) and *Phalaenopsis* (Lee et al., 2013). For the study of HRGP function, an inhibitor of HRGP biosynthesis, 3,4-dehydro-L-proline (3,4-DHP), was applied to alter the contents of HRGPs in cell walls of orchid embryos to identify any significant consequences of altering HRGPs on symbiotic germination.

## Materials and Methods

### Symbiotic Germination

*Dendrobium officinale* plants were cultivated in the greenhouse at the Menghai experimental station, Institute of Medicinal Plant Development, Chinese Academy of Medical Sciences and Peking Union Medical College, Xishuangbanna, Yunnan, China. The flowers were pollinated by hand, and the capsules were collected just prior to dehiscence at 180 days after pollination. The capsules were surface sterilized with 1% sodium hypochlorite solution for 20 min in the laboratory. After surface sterilization, the capsules were cut open, and the seeds were taken out and placed onto the surface of 2 cm × 2 cm sections of nylon cloth within a 9 cm diameter Petri dish containing 20 mL sterile oatmeal agar (OMA: oat 2.5 g L^-1^, agar 12 g L^-1^, the pH measured at 5.2 prior to autoclaving). The OMA medium was autoclaved at 101.33 kPa and 121°C for 20 min. About 100 seeds were sown onto each nylon cloth section, with four nylon cloth sections per Petri dish. The mycorrhizal fungal isolate (*Tulasnella* S6 strain) was incubated on fresh Potato Dextrose Agar (PDA: infusion from potato 200 g L^-1^, dextrose 20 g L^-1^, agar 15 g L^-1^, the pH measured at 5.6 prior to autoclaving) in darkness at 25°C for 10 days, then the actively growing mycelia from the colony margin were severed and used as the fungal inoculum. Each seed-containing plate was inoculated with five pieces (0.5 cm^3^) of fungal inoculum, the plates without fungal inoculum served as the control. Plates were sealed with PARAFILM^®^. Thirty replicate plates were maintained in the growth room under a 12/12-h photoperiod at 30 μmol m^-2^ s^-1^ (daylight fluorescent tubes FL-20D/18, 20 W). The plates were observed and recorded under a dissecting stereomicroscope every week after inoculation. Seed germination and the growth of protocorms were scored as defined by Stewart et al. (2003). Germination was defined as emergence of the embryo from the seed coat, i.e., stage 2.

### Asymbiotic Germination

After surface sterilization, the capsules were cut open, and the seeds were taken out and placed onto 20 ml modified Murashige and Skoog (MS) medium (Murashige and Skoog, 1962) in a 9 cm diameter Petri dish. The modified MS medium contained 1/2 strength of macroelements with full strength of microelements, 2 mg L^-1^ glycine, 0.5 mg L^-1^ niacin, 0.5 mg L^-1^ pyridoxine HCl, 0.1 mg L^-1^ thiamine, and 100 mg L^-1^ myo-inositol, 20 g L^-1^ sucrose, and 7 g L^-1^ agar. The pH was adjusted to 5.7 before autoclaving at 101.33 kPa and 121°C for 20 min. The cultures were maintained in the growth room under a 12/12-h photoperiod at 30 μmol m^-2^ s^-1^ as described above. After seed germination, developing protocorms were collected for the immunofluorescence labeling and real-time PCR experiments.

### Histological Study

The seeds and mycorrhizal protocorms were fixed in a solution of 2.5% glutaraldehyde and 1.6% paraformaldehyde in 0.05 M phosphate buffer (pH 6.8) for 4 h at room temperature. After fixation, the samples were dehydrated using an ethanol series, and embedded in Technovit 7100 (Kulzer and Co., Germany) as described by Yeung and Chan (2015). Serial, 3 μm-thick sections were cut with glass knives using a Reichert-Jung 2040 Autocut rotary microtome. These sections were stained with Periodic acid–Schiff’s reaction for total insoluble carbohydrates, and counterstained with either 0.05% (w/v) toluidine blue O (TBO) in benzoate buffer for general histology or 1% (w/v) amido black 10B in 7% acetic acid for protein (Yeung, 1984). The sections were examined and the images were captured digitally using a CCD camera attached to a light microscope (Axio Imager A1, Carl Zeiss AG). More than 100 different protocorms of each developmental stage were studied.

### Identification of Extensin-Like Genes From Expressed Sequence Tags Database

Four putative genes coding for extensin-like genes were selected from the expressed sequence tags database (Chen et al., 2017), and were compared with the NCBI non-redundant protein database (Nr) using BLASTX after translating DNA sequences into the respective amino acid sequences. The GenBank accession numbers were KX906493, KX906494, KX906495, and KX906496.

### RNA Extraction and Real-Time PCR

Total RNA was extracted from mature seeds, symbiotically and asymbiotically germinated protocorms using RNeasy Plant Mini Kit (Qiagen, Hilden, Germany) according to the manufacturer’s instructions. RNA samples were treated with RQ1 DNase (Invitrogen, United States) to remove DNA remnants, then underwent synthesis of the first cDNA strand by using the Prime Script RT reagent Kit (TaKaRa Bio, Japan). Primers for real-time PCR were designed by using Primer Premier 5.0 (Premier Biosoft, India) and the actin gene was used as an internal quantification standard (**Supplementary Table S1**). Each real-time PCR experiment contained 7.5 μL of SYBR Premix Ex Taq II (TaKaRa Bio), 1.5 μL of cDNA, and 0.3 μL primers, and water was added to 15 μL. For each real-time PCR, each sample was analyzed in three biological replicates with three technical replicates using the Light Cycler 480 II Real-Time PCR System (Roche, Switzerland) with its relative quantification program. The parameters of reactions were consisted of an initial denaturation at 95°C for 30 s, then 40 cycles of 95°C for 5 s, and 60°C for 30 s. The 2^-ΔΔCt^ method was used for evaluating gene expression. The data were statistically analyzed using ANOVA followed by Fisher’s protected least significant difference test.

### Immunodot Blot Assay of HRGPs

A set of JIM antibodies (JIM 11, JIM 12, JIM13, JIM14, JIM15, JIM16, and JIM20) was obtained from PlantProbes (Leeds, United Kingdom) to test the presence of HRGPs. In a preliminary survey using the immunodot blot assay, JIM 11 gave the most intense staining in the zygotic embryo. To evaluate if JIM11 recognized an epitope of extensin, another monoclonal antibody to extensin, LM1 (Smallwood et al., 1995) from PlantProbes, was also tested. JIM11 staining mirrored that of LM1 (**Supplementary Figure S1**), confirming that JIM11 recognized extensin. Therefore, we used JIM11 as the cell wall marker in the experiment of immunofluorescence labeling of HRGPs. The procedure for immunodot blot assay of HRGPs has been described by Lee et al. (2013). Briefly, samples of equal fresh weight (200 mg) were collected and ground into fine powders in liquid nitrogen. Proteins were extracted using 0.7 mL extraction buffer [100 mM Tris, 900 mM sucrose, 10 mM ethylene diamine-tetra-acetic acid (EDTA), 100 mM KCl and 0.4% (v/v) mercapto-ethanol, pH 8.8] and 0.7 mL of Tris-saturated phenol (pH 8.8). After centrifugation at 8,000 rpm for 5 min at 4°C, the supernatant was collected for protein precipitation. The proteins were precipitated by the addition of five volumes of 0.1 M ammonium acetate (in 100% methanol) to the phenol phase, and left at -20°C overnight. The precipitate was centrifuged at 16,000 × *g* for 20 min at 4°C. The pellet was dissolved in rehydration buffer [8 M urea, 2% CHAPS, 2% Triton X-100, 50 mM 1,4-dithiothreitol (DTT)]. Samples were boiled at 96°C for 5 min and were equally spotted on a nitrocellulose membrane by a micropipette (as 5 μL drops). The membrane was air-dried for 1 h and blocked in PBS buffer containing 5% (w/v) milk powder and 0.5% BSA for 1 h, followed by labeling with the primary monoclonal antibodies, JIM11 and LM1. The primary antibodies were diluted 1:1000 in PBS containing 1% BSA. After three washes with PBST (PBS buffer containing 0.2% Tween 20) for 10 min, the membrane was probed with a 1:2500 dilution of the secondary antibody, horseradish peroxidase (HRP)-conjugated anti-rat IgG at room temperature for 1 h. After the final wash with PBST, the detection of signal was performed by adding chemiluminescent HRP substrate (WBKL S0500, Millipore, Billerica, MA, United States) and captured by a luminescent image analyzer, LAS-4000 (FUJIFILM, Japan). Three biological replicates have been performed in the assay of immunodot blot of HRGPs.

### Immunofluorescence Labeling of JIM11

For immunofluorescence labeling of HRGPs, samples were collected and fixed in 4% (v/v) paraformaldehyde in stabilizing buffer MSTB [50 mM piperazine-N, N′-bis(2-ethanesulfonic acid) (PIPES), 5 mM MgSO_4_.7H_2_O, 5 mM ethylene glycol-bis(2-aminoethylether)-*N, N, N*′, *N*′-tetraacetic acid (EGTA), pH 6.9] and left overnight at 4°C. Samples were dehydrated in an ethanol series (30, 50, 70, 90, and 100%) and placed in pure acetone. The samples were infiltrated gradually with a graded series of 100% acetone to Technovit 8100 (3:1, 1:1, and 1:3; Kulzer and Co., Wehrheim, Germany), followed by two changes of pure Technovit 8100 (Yeung and Chan, 2015). The resin was polymerized at 4°C and 3 μm thick sections were cut, placed on a drop of water on a slide and dried at room temperature. Sectioned samples were rehydrated in PBS, pH 7.3, for 5 min followed by blocking in a 2% bovine serum albumin (BSA) in PBS for 5 min. The sections were labeled with primary monoclonal antibody, JIM11, and left overnight at 4°C. The primary antibodies were diluted 1:20 in PBS containing 1% BSA. After washing in PBS three times (5 min each time), sections were incubated with a 1:20 dilution of the secondary antibody (anti-rat IgG-FITC, F6258, Sigma) in PBS containing 1% BSA for 1 h in the dark, followed by three washes in PBS (10 min each time). For quenching the tissue autofluorescence, the sections were stained with 0.01% TBO in PBS for 1 min. After washing in PBS three times (5 min each), sections were mounted in medium containing an anti-fade mounting reagent (VECTASHIELD^®^ Mounting Medium, Vector Laboratories, Inc., Burlingame, CA, United States) before observation. Controls were prepared by incubating with the blocking solution instead of the primary antibodies. The sections were examined and the images were captured using a LSM510 META confocal laser-scanning microscope (Carl Zeiss AG). For FITC detection, the 488 nm laser was used for excitation, and the emission filter was set to detect in the 500–530 nm range. To detect the autofluorescence, the 488 nm laser was also used for excitation, and the emission filter in the 565–615 nm range was used for detection. Three biological replicates have been performed in the experiment of immunofluorescence labeling, and more than thirty different protocorms were observed in each replicate.

### Immunogold Observation

Symbiotic protocorms at stage 3 were collected and fixed by high-pressure freezing fixation in a high-pressure freezer (Leica EM PACT2). The fixed protocorms were subjected to freeze substitution in ethanol (containing 0.2% glutaraldehyde and 0.1% uranyl acetate) in a Leica Automatic Freeze-Substitution System, then embedded in London Resin White methacrylate resin (London Company, Basingstoke, United Kingdom). Ultrathin sections (70–90 nm) were cut with a diamond knife on a Leica Reichert Ultracut S (Leica Microsystems GmbH^1^) and placed on formvar-coated nickel grids. For transmission electron microscopy (TEM) observation, the immunological staining was performed as previously described (Van Aelst and Van Went, 1992) with minor modifications. The sections were incubated in 3% normal goat serum and 0.5% BSA in PBST for 10 min at room temperature. Grids were then transferred to another drop of PBST containing the JIM11 antibody (1:200) and 0.5% BSA for 1 h at room temperature. After washing thoroughly with PBST, the sections were then treated for 20 min at room temperature with goat anti-mouse colloid gold conjugates (18 nm; RPN422 Auro-Probe EMGAR G18, Amersham), diluted 1:20 in PBST. After a further wash with PBST and distilled water, the sections were counterstained with uranyl acetate, followed by lead citrate. Controls were made to confirm the specificity of the immunological staining procedure, including (1) the incubation with pre-immune mouse IgG (Vector Laboratories, Inc.) and (2) the incubation with colloidal gold-conjugated anti-mouse IgG, omitting the first antibody. A Philip CM 100 transmission electron microscope (FEI Company^2^) at 80 kV was used for observation. At least three protocorms and more than 30 sections for each time point were examined.

### Treatment With 3,4-Dehydro-L-Proline (3,4-DHP)

The role of HRGPs in the symbiotic germination of *D. officinale* was investigated using 3,4-DHP, an inhibitor of prolyl hydroxylase to alter the structure of HRGPs in cell walls (Xu et al., 2011). In symbiotic germination, 200 μM of 3,4-DHP (Sigma-Aldrich Co.) was added to the OMA medium with fungal inoculum. Plates without 3,4-DHP treatment were used as controls. The seed germination experiments were processed as described above. Germination was defined as emergence of the embryo from the seed coat. After 3 weeks of culture, the germination percentage was calculated as the percentage of the number of seeds germinated among the total countered number of seeds with embryos. At the same time, the expression of JIM11 antigen in 3,4-DHP-treated cultures was examined by fluorescence labeling as described above. Experiments were performed using a completely randomized design. Twelve plate replicates were examined for each treatment. The data were analyzed statistically using ANOVA followed by Fisher’s protected least significant difference test.

## Results

### Symbiotic Germination

**FIGURE 1:**
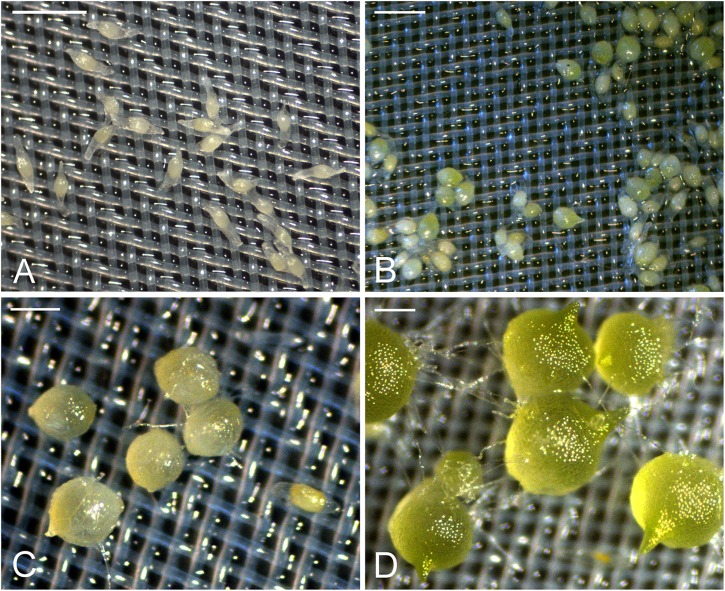
The successive developmental stages of *D. officinale* from seed germination to protocorm formation in symbiotic culture. **(A)** Stage 0, the embryos enclosed by the intact seed coats in the first week of inoculation. Scale bar = 0.5 mm. **(B)** Stage 1, swollen seeds after 1 week of inoculation. Scale bar = 0.5 mm. **(C)** Stage 2, globular protocorm rupturing the seed coat after 2 weeks of inoculation. Scale bar = 1 mm. **(D)** Stage3, green protocorm with the shoot tip and rhizoids after 3 weeks of inoculation. Scale bar = 1 mm.

In the first week of inoculation, the embryos had absorbed water and became slightly swollen, but the seed coats remained intact (stage 0) (**Figure 1A**). After 1 week of inoculation, most embryos had become swollen and some embryos began to turn light-green (stage 1) (**Figure 1B**). After 2 weeks of inoculation, the embryos continued to swell and then ruptured the seed coat (stage 2) (**Figure 1C**). After 3 weeks of inoculation, more than 70% of inoculated seeds had germinated. The embryos developed further and resulted in the formation of green protocorms (stage 3). At this stage, a shoot tip became visible at one end of a protocorm, and numerous rhizoids formed at the opposite end (**Figure 1D**).

### Histological Study

**FIGURE 2:**
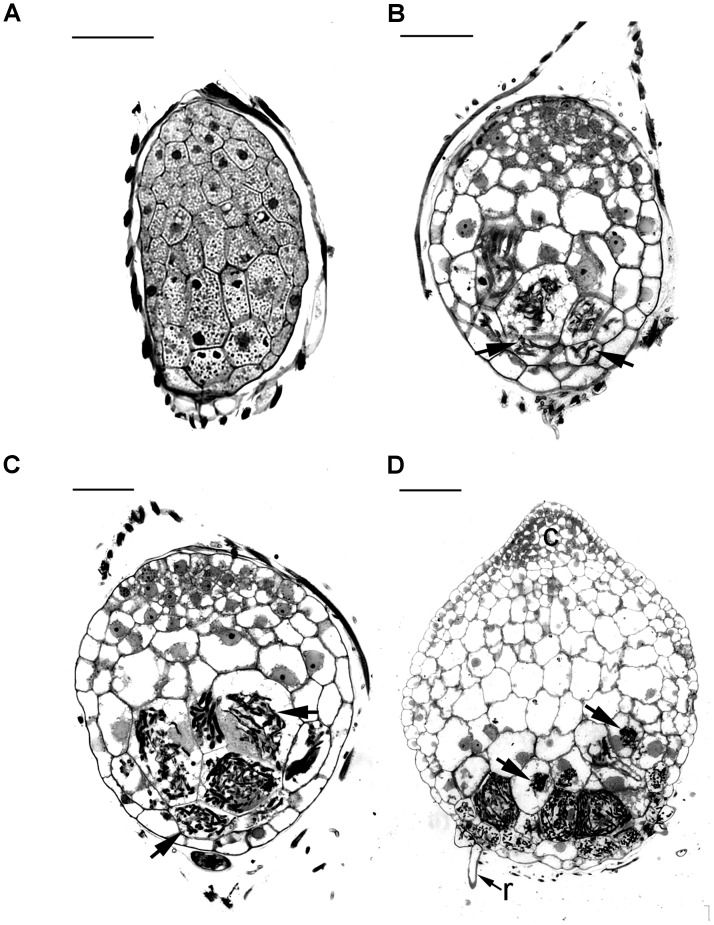
Light micrographs of sections showing the successive developmental stages of *D. officinale* from seed germination to protocorm formation in symbiotic culture. **(A)** Stage 0, the uncolonized embryo covered by a thin seed coat in the first week of inoculation. The embryo cells filled with storage products, such as protein and lipid bodies. Scale bar = 50 μm. **(B)** Stage 1, the infected embryo after 1 week of inoculation. Fungal hyphae have colonized the embryo and formed the pelotons (arrows) within embryo cells. Larger vacuoles make the embryo cells expand and the storage nutrients disappear from most embryo cells. Scale bar = 50 μm. **(C)** Stage 2, the enlarged embryo with the rupture of the seed coat after 2 weeks of inoculation. The pelotons (arrows) locate in outer cortical cells and inner cortical cells of the spherical protocorm. Scale bar = 50 μm. **(D)** Stage3, protocorm with the first crest and rhizoids after 3 weeks of inoculation. At this stage, the pelotons (arrows) are digested in the inner cortical cells. c, crest; r, rhizoid. Scale bar = 100 μm.

**FIGURE 3:**
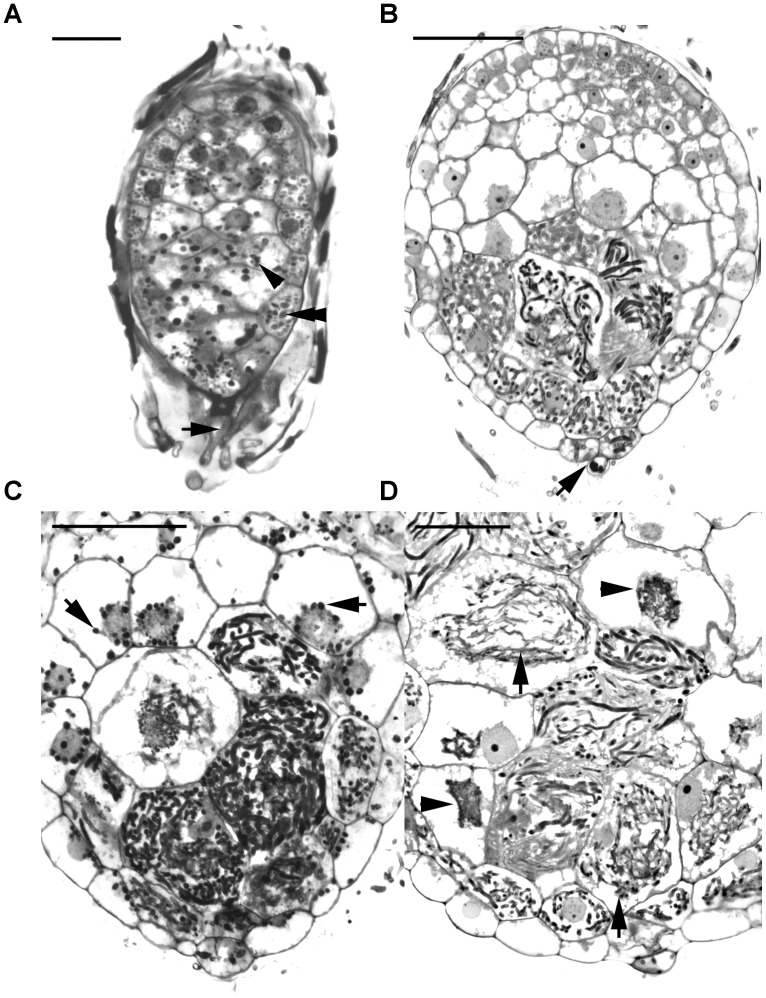
Longitudinal sections of developing protocorms from symbiotic cultures showing the cells with the development and degeneration of fungal hyphae. **(A)** In the first week of inoculation, the embryo has become slightly swollen, and the fungal hyphae (arrow) have just touched the suspensor end of embryo. Within the embryo cells, the protein bodies are degenerating (double arrowhead), and the starch grains appear (arrowhead). Scale bar = 50 μm. **(B)** Fungal hyphae have penetrated the embryo through the suspensor end (arrow) and colonized the middle and basal cortical cells of the enlarged embryo at stage 1. Scale bar = 50 μm. **(C)** In a developing protocorm at stage 2, the peloton hyphae are primarily found in the middle and basal cortical cells. In the infected cortical cells, starch grains were depleted, while in the top portion of the protocorm, chloroplasts had appeared and starch grains (arrows) were noticeable within the cytoplasm. Scale bar = 50 μm. **(D)** As the protocorm further grows up (stage3), there are a number of collapsing peloton hyphae (arrows) and degenerated hyphal clumps (arrowheads) in the cortical cells. Scale bar = 50 μm.

Before the invasion by fungal hyphae, the uninfected embryos, covered by a thin layer of seed coat, were only ten to eleven cells long and six to seven cells wide (**Figure 2A**). A gradient of cell size was observed in the embryos with smaller cells located at the chalazal end. Within the embryo cells, protein and lipid bodies were present. In the first week of inoculation, embryos became slightly swollen due to the uptake of water, and the fungal hyphae congregated at the suspensor end of the embryos (**Figure 3A**). At this time, storage products, i.e., protein and lipid bodies began to break down, while starch grains appeared and tended to congregate near the nuclei. After 1 week of inoculation, the embryos had enlarged by vacuolization and the storage products, i.e., protein and lipid bodies had disappeared from most cells (**Figure 2B**). At this stage, fungal hyphae had penetrated the embryos through their suspensor end. The large, basal cells of the swollen embryos became colonized by hyphae (**Figure 3B**). Cell divisions occurred at the apical (chalazal) end of the embryos, generating a zone of meristematic cells. After 2 weeks of inoculation, the embryos continued to enlarge, rupturing the seed coat. This resulted in the formation of spherical protocorms (**Figure 2C**). The mycorrhizal hyphae were found mainly in the outer and inner cells at the basal (suspensor) end of the protocorms (**Figure 3C**). After 3 weeks of inoculation, protocorms developed further with the formation of the first crest at the apical end and the rhizoids at the basal end (**Figure 2D**). At this stage, collapsing peloton hyphae and degenerated hyphal clumps were present in the basal cells of developing protocorms (**Figure 3D**). During symbiotic germination, mycorrhizal hyphae were confined to the basal end of the protocorm and not present in the developing shoot pole.

### Expression Patterns of *D. officinale* Extensin-Like Genes

**FIGURE 4:**
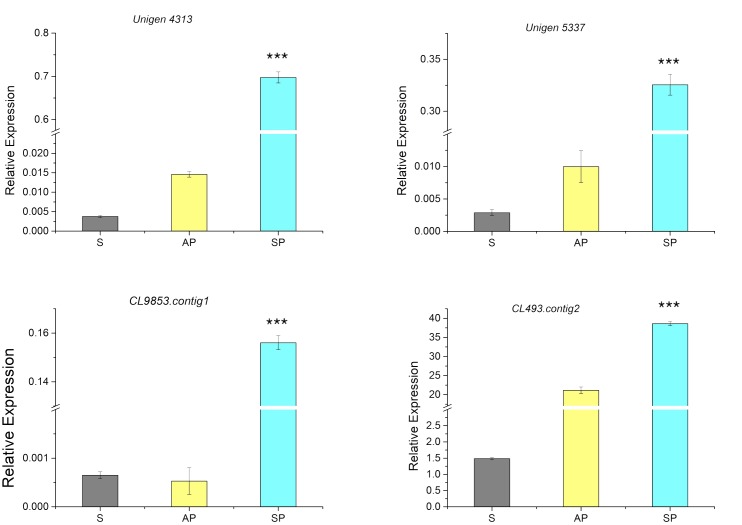
Expression of genes coding for extensin-like genes in the mature seeds (S), symbiotically (SP), and asymbiotically (AP) germinated protocorms measured by real-time PCR. ^∗∗∗^Indicates significant difference at *P* < 0.001 probability.

From the RNA-seq database of the symbiotic protocorm (Chen et al., 2017), we selected sequences coding four different extensin-like proteins (**Table 1**), i.e., *Unigene5337*, *CL9853*, *CL493.contig2* and *Unigene4313*. Real-time PCR of these four genes performed to verify their expression in mature seeds and symbiotically and asymbiotically germinated stage 3 protocorms (**Figure 4**). The four extensin-like genes showed low expression levels in mature seeds and asymbiotically germinated protocorms (except for *CL493.contig2*), while high expression levels were detected in symbiotically germinated protocorms.

**Table 1:** *D. officinale* extensin-like genes investigated by real-time PCR.

Sequence code	GenBank accession	Blastx result	Reference gene accession	Query coverage	*E*-value	Conserved domain
CL9853.contig1	KX906495	Extensin2 like protein	XP_008791833	52%	7.00E-50	Pollen proteins Ole e I like (pfam01190)
Unigene4313	KX906494	Extensin2 like isoform X1	XP_012481562	46%	0.030	Extensin_2 (pfam04554)
Unigene5337	KX906496	LLR extensin like protein	XP_019707248	78%	0	Leucine-rich repeat (pfam13855)
CL493.contig2	KX906493	Extensin like	XP_020242813	88%	0.007	–


### Immunodot Blot, Immunofluorescence, and Immunogold Localization of JIM11 Epitope in Symbiotic Germination

**FIGURE 5:**
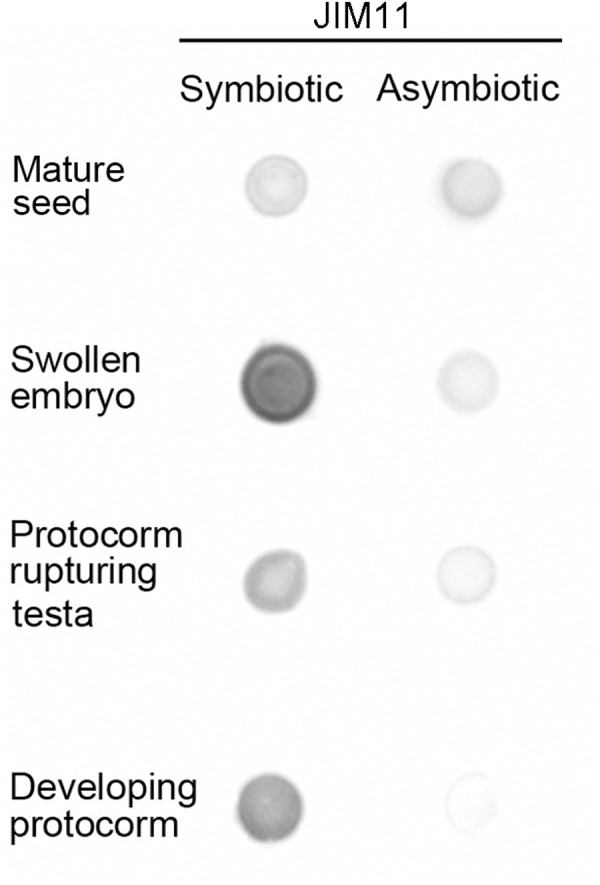
Semi-quantitative analysis by immunodot blots of the relative abundance of JIM11 epitope in extracts prepared from the mature seeds and developing protocorms in the symbiotic and asymbiotic cultures.

For the assay of immunodot blot in the symbiotic cultures, a moderate intensity of JIM11 staining could be observed in the mature seeds. The signal of JIM11 in swollen embryos (stage 1) was relatively high, while the signal intensities in the developing protocorms decreased after germination (stages 2 and 3). On the contrary, in asymbiotic cultures, the signals of JIM11 in swollen embryos and developing protocorms were much lower as compared with those stages in symbiotic cultures (**Figure 5**).

**FIGURE 6:**
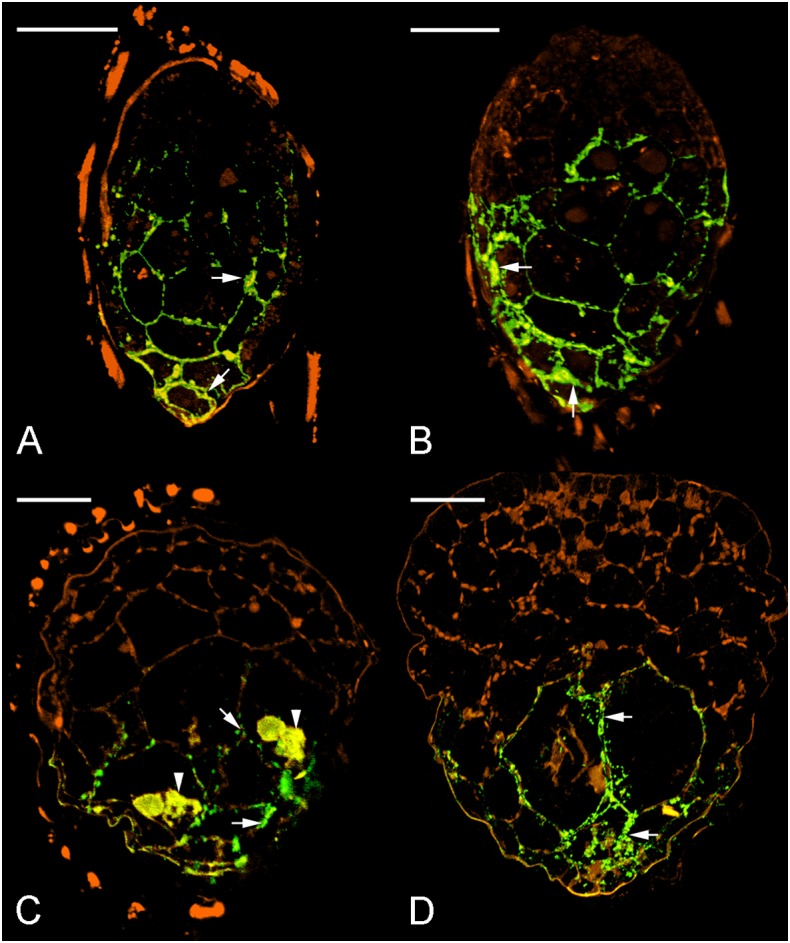
Immunofluorescence localization of JIM11 epitope during symbiotic seed germination of *D. officinale*. **(A)** The uncolonized embryo with strong signals of JIM11 epitope (green color, excitation 488 nm and emission 500–530 nm). The signals (arrows) are present mainly in the walls of the middle and basal regions of the embryo. The orange color indicates the autofluorescence (excitation 488 nm and emission 565–615 nm). Scale bar = 50 μm. **(B)** After 1 week of inoculation, the infected embryo showing strong signals of JIM11 epitope (arrows) in the walls of the middle and basal regions of the swollen embryo. Scale bar = 50 μm. **(C)** After 2 weeks of inoculation, the enlarged embryo with the rupture of the seed coat showing prominent signals of JIM 11 epitope in the pelotons (arrowheads) and the walls of colonized cells (arrows) in the basal protocorm. Scale bar = 50 μm. **(D)** After 3 weeks of inoculation, the signals of JIM 11 epitope (arrows) are mainly observed in the walls of colonized cells of the developing protocorm. Scale bar = 100 μm.

Since dot blot results represented the total contents of JIM11 from protocorms at different stages of germination, a qualitative comparative overview of immunolabeling of JIM11 epitope was carried out and the result is summarized in **Table 2**. In the uninfected seeds, conspicuous fluorescence signals of JIM11 epitope were observed, with a stronger signal in the larger cells near the basal (suspensor) end, and weaker signals at the apical (chalazal) end of the embryos (**Figure 6A**). After 1 week of inoculation, the basal end of embryos had been infected by the hyphae, and a strong signal was detected mostly at the middle and basal cells of enlarging embryos (**Figure 6B**). Fluorescent signals could also be found in adjacent uninfected cells in the basal end of protocorms, while no signal was detected in cells of the future shoot pole (**Figure 6B**). As protocorm continued to develop, the signal was concentrated mainly in the colonized cortical cells at the basal end. In the infected cells, the signals were present in the walls and intracellularly associated with hyphae and the pelotons (**Figures 6C,D**). In the negative staining control, no signal can be detected in the embryo after incubation without the primary antibody of JIM11 (**Supplementary Figure S2**).

**Table 2:** The intensity evaluation of immunofluorescence labeling with JIM11 antibody during the formation of protocorm of *D. officinale* in symbiotic culture.

Developmental stages	Embryo regions	JIM11 signal intensity
Stage 0	Apical region	-
	Middle region	++
	Basal region	++
Stage 1	Apical region	-
	Middle region	+++
	Basal region	++++
Stage 2	Apical region	-
	Middle region	+
	Basal region	+++
Stage 3	Apical region	-
	Middle region	+
	Basal region	+++


*Increasing intensity was evaluated as: - (no signal); ±(very weak); + (weak); ++ (intermediate); +++ (strong); ++++ (very strong).*

**FIGURE 7:**
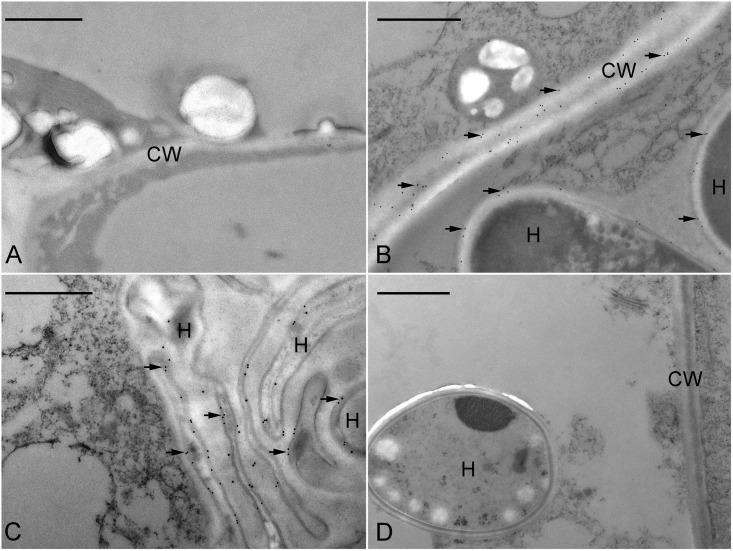
Immunogold staining of JIM11 epitope in the symbiotic protocorms of *D. officinale*. **(A)** In the apical portion of the protocorm, no immunogold particles were observed. Scale bar = 1 μm. **(B)** TEM of a basal portion of protocorm cell with fungal hyphae (H). Labeling occurred in the host cell wall (CW) and the interfacial matrix (arrowhead) or fungal cell wall. Scale bar = 1 μm. **(C)** TEM of a portion of protocorm cell with collapsed fungal hyphae (H). Scale bar = 1 μm. **(D)** The control staining. Thin sections were incubated with the mouse pre-immune IgG instead of JIM11 antibody. Immunogold particles were absent in these treatments. Scale bar = 1 μm.

In order to determine the precise localization of JIM11 epitope within the infected cells of symbiotic protocorms at stage 3, the immunogold staining was used. No immunogold particles were detected in the cell wall at the apical part of protocorm (**Figure 7A**). Immunogold particles were observed in the colonized cortical cells at the basal part of protocorm, particularly where they were deposited in the protocorm cell wall (**Figure 7B**). A few immunogold particles were observed in the interfacial matrix near the fungal cell wall (**Figure 7B**), as well as around the collapsed fungal hyphae (**Figure 7C**). The control with the incubation with pre-immune mouse IgG or the omission of JIM11 antibody showed no immunogold particles in any infected cells (**Figure 7D**).

### Effect of 3,4-DHP on Symbiotic Germination

**FIGURE 8:**
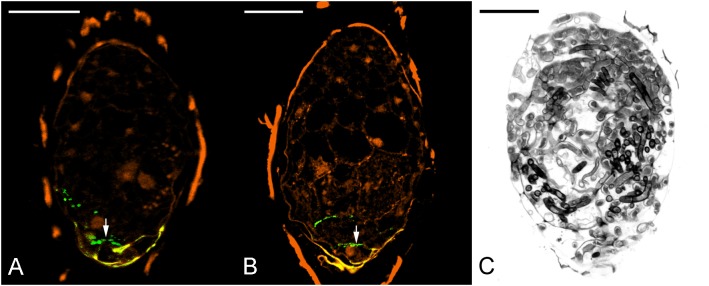
The effect of 3,4-DHP on the localization of JIM11 epitope during symbiotic seed germination of *D. officinale*. **(A)** After 3 weeks of inoculation, most embryos did not enlarge, and weak signals of JIM11 epitope (arrow) are observed in the walls of basal end of the embryo. Scale bar = 50 μm. **(B)** After 3 weeks of inoculation, the infected embryo showing weak signals of JIM11 epitope (arrow) in the walls of basal end of the swollen embryo. Scale bar = 50 μm. **(C)** Most seeds in the treatment with 3,4-DHP are unable to germinate, and the severe fungal invasion in the ungerminated seeds could be commonly observed. Scale bar = 50 μm.

To evaluate the effect of 3,4-DHP on symbiotic germination, 3,4-DHP was added to the OMA medium with fungal inoculum. After 3 weeks of culture, a remarkable decreased in germination percentage was observed as compared with the untreated control culture (**Table 3**). To evaluate the effect of 3,4-DHP on the distribution and localization of HRGPs in developing protocorms during symbiotic germination, immunolabeling with JIM11 antibody was carried out on seeds cultured on the symbiotic germination medium supplemented with 3,4-DHP. After 3 weeks of culture, very low expression of the JIM11 epitope was detected in the ungerminated embryos treated with 3,4-DHP (**Figure 8A**). With the 3,4-DHP treatment, only a few embryos enlarged and reached stage 1 (**Table 3**), and a very weak labeling with the JIM11 antibody was observed in the walls of basal region of protocorms (**Figure 8B**). In the ungerminated protocorm, severe fungal invasion could be commonly observed (**Figure 8C**). The hyphae could be found throughout the entire protocorm and a well-defined zone of meristematic cells located at the future shoot pole was absent.

**Table 3:** Effect of 3,4-DHP on symbiotic seed germination of *D. officinale*.

Treatment	Germination	Developmental stages			
		
	(%)	0	1	2	3
Control	92.9^a^	0	7.1	20.4	72.5
200 μM 3,4-DHP	1.8^b^	61.8	36.4	1.8	0


*Means having the same letter in a column are not significantly different at 5% level by Fischer’s protected LSD test. Data were scored after 3 weeks of culture. Germination was defined as emergence of the embryo from the seed coat, i.e., stage 2.*

## Discussion

In orchids, the establishment of seedlings requires the formation of protocorms. The protocorm is a post-embryonic structure from which a shoot and a root subsequently differentiate (Arditti and Krikorian, 1996). In symbiotic germination, compatible fungi colonize orchid seeds and provide nutrients for the formation of protocorms (Rasmussen, 1995; Peterson et al., 1996; Selosse and Martos, 2014).

Hydroxyproline-rich glycoproteins are abundant cell wall components with different functions (Berger et al., 1994; Cassab, 1998). They have been shown to involve in symbiotic interactions (Berry et al., 2002; Olsson et al., 2002; Rathbun et al., 2002) and in plant defense (Davies et al., 1997; Raggi, 2000). In this study, the results of immunodot blot analysis indicate that HRGPs are present in mature seeds and mycorrhizal protocorms of *D. officinale*. The immunofluorescence signals of JIM11 epitopes are primarily present in the suspensor end of uncolonized and colonized embryos (**Figure 6**). Four extensin-like genes are up-regulated supporting the immunolocalization studies (**Figure 4**).

Successful penetration of fungal hyphae into protocorms is a key step in establishing mycorrhizal association. The presence of JIM11 epitope at the suspensor end of the embryo may serve as a possible recognition site of fungal colonization. Previous reports have indicated that the suspensor end of an orchid embryo is one of the main sites for the penetration of fungal hyphae (Peterson and Currah, 1990; Rasmussen, 1990). In this study, by 5 days after inoculation, fungal hyphae have reached the suspensor end of *Dendrobium* embryo (**Figure 3A**) and have penetrated into the basal parenchyma cells through the degenerated suspensor after 1 week of inoculation (**Figure 3B**). It is well established in orchid embryos, a distinct cuticle is present over the entire embryo proper surface but is absent from the suspensor cell wall (Yeung et al., 1996; Lee et al., 2006; Lee and Yeung, 2010). Although the suspensor has degenerated as the seed matured, the presence of primary wall not covered by a cuticle makes the suspensor end a vulnerable or a predetermined site for fungal penetration.

The signals for JIM11 epitope become stronger in the *Dendrobium* protocorms as colonization begin to take hold (**Figure 6**). On the contrary, as compared to asymbiotic germination control, JIM11 epitope signals remain weak upon germination (**Supplementary Figure S3**). In parsley, the expression of HRGP transcripts is much higher in mycorrhizal roots than those in the uncolonized control (Franken and Gnadinger, 1994). In maize root, the activation of HRGP transcripts is induced by arbuscular mycorrhizal fungi, and this response is restricted to mycorrhizal tissues (Balestrini et al., 1997). In this study, four extensin-like genes are up-regulated primarily in symbiotically germinated protocorms (**Figure 4**). This result supports the work by Balestrini et al. (1997) and Xie et al. (2011) that extensin-like genes are involved in the interactions between plants and microorganisms, and symbiotic association. More importantly, the up-regulation of extensin-like genes corroborates the fluorescent staining patterns of JIM11. Our results suggest that mycorrhizal colonization may induce *de novo* synthesis of HRGPs in basal cells of *Dendrobium* protocorms in preparation for the colonization process.

During symbiotic germination, fungal colonization is restricted to the basal portion (suspensor end) of a protocorm (**Figures 2B–D**). Fluorescent signals of JIM11 epitope are primarily located in the walls and in association with the hyphae and the pelotons of colonized cells. It is important to note that fluorescent signals can also be found in adjacent uncolonized cells of a protocorm, while no signals are detected in the apical end (**Figures 6B–D**, **7A**). The accumulation of HRGPs in the intracellular interfaces between hyphae and peri-fungal host membrane has been reported in various plant/arbuscular mycorrhizal fungus combinations (Bonfante-Fasolo et al., 1991; Bonfante, 2001; Balestrini and Bonfante, 2005). In this study, the precise localization of JIM11 epitope within symbiotic protocorms is further examined by immunoelectron microscopy. In the colonized cells, immunogold particles were primarily observed in the cell wall as well as the interfacial matrix near the fungal cell wall (**Figure 7B**). The formation of plant-fungal interfaces is crucial for establishing the partnership in mutualistic relationships that allows a two-way exchange of signal molecules and nutrients (Smith and Smith, 1990). Our results suggest that HRGPs are integral parts of the interface that is essential for the establishment of symbiotic association between orchid protocorms and mycorrhizal fungi.

Furthermore, it is noteworthy that the cross-linking of HRGPs such as extensin through peroxidation plays an important role in strengthening of plant cell wall (Jackson et al., 2001). In pathogenic interactions, the rapid insolubilization of extensin is an essential component of the plant’s primary defensive reaction to microbial attack (Lamb and Dixon, 1997). But these defense responses seem to be weakly or transiently activated as compared to pathogenic interactions. In arbuscular mycorrhizal symbiosis, the penetration of fungal hyphae induces HRGPs-encoding genes in mycorrhizal tissues (Balestrini et al., 1997). In the legume-rhizobium symbiosis, the expression of extensin genes in root hairs and nodules has been proposed to remodel plant cell wall architecture and to limit the bacterial growth in infection thread (Arsenijevic-Maksimovic et al., 1997; Wisniewski et al., 2000). In this study, the extensin-like genes are highly up-regulated in the symbiotic protocorms as compared to the asymbiotic protocorms (**Figure 4**), and JIM11 epitope are localized in the cells containing fungal hyphae (**Figures 6**, **7**). The results from our studies indicated that HRGPs may be essential for limiting the fungal spread inside the protocorms and modulating the accommodation process of the mycorrhizal fungi inside the basal cells of protocorms, allowing the formation of a shoot apical meristem at the apical end of protocorm.

It is clear in this study that HRGPs are not present in the rapidly dividing cells where the shoot apical meristem will subsequently develop. Since HRGPs may serve to strengthen cell walls, their absence will not hinder rapid cell division leading to SAM formation. It is interesting to note that HRGPs are present in the maize root meristem cell walls (Balestrini et al., 1994). Their presence may serve to protect the root meristem from infection, enabling the continual growth of the root. Future studies will further our insight into the role of HRGPs in meristem development and function in orchids.

The importance of HRGPs is clearly demonstrated by 3,4-DHP treatment. In symbiotic cultures treated with 3,4-DHP, little JIM11 epitopes were present in these colonized embryo cells (**Figures 8A,B**), and severe fungal invasion was observed (**Figure 8C**). The absence of HRPGs alters the internal regulatory process resulting in the uncontrolled growth of the hyphae, leading to developmental arrest and death of protocorms. Judging from the inhibitor studies, HRPGs may have a defensive role to play as they serve to separate the colonized cells from apical cells destined to form the shoot apical meristem.

## Conclusion

In conclusion, this is the first report that shows the accumulation of HRGPs as recognized by JIM11 epitope, in the mycorrhizal protocorm in response to colonization by symbiotic fungi. The treatment with 3,4-DHP inhibits symbiotic germination and the accumulation of HRGPs in colonized cells of seeds. The presence of HRGPs in the basal region of protocorm appears to be essential for the establishment of a symbiotic relationship between *D. officinale* and *Tulasnella*. With the accomplishment in genome sequencing of *D. officinale* (Yan et al., 2015), future molecular genetics studies will provide new insight into the regulatory process for orchid symbiosis.

## Author Contributions

Y-IL and S-XG conceived the study. Y-IL, S-XG, X-MC, Y-YL, and A-RW designed the study. XZ performed sequence analyses. Y-YL and YZ performed qPCR experiments. Y-IL, Y-YL, and EY performed histological and immunohistochemical studies. Y-HC and Y-YL performed immunodot blots experiments. Y-IL, S-XG, EY, and Y-YL wrote the paper. All authors read and approved the final manuscript.

## Conflict of Interest Statement

The authors declare that the research was conducted in the absence of any commercial or financial relationships that could be construed as a potential conflict of interest.

## Footnotes

1 http://www.leica-microsystems.com/

2 http://www.feicompany.com

## Supplementary Material

FIGURE S1 Semi-quantitative analysis by immunodot blots of the relative abundance of LM1 epitope in extracts prepared from the mature seeds and developing protocorms in the symbiotic and asymbiotic cultures.

FIGURE S2 In the negative control of immunofluorescence localization of JIM11 epitope, no signal can be detected in the enlarged embryo after incubation without the primary antibody of JIM11. Scale bar = 50 μm.

FIGURE S3 In the asymbiotic germination culture, **(A)** after 3 days of culture, the signals of JIM11 epitope (green color) were located in the walls of the middle and basal regions of the imbibed embryo, scale bar = 50 μm; **(B)** after 2 weeks of culture, a few signals of JIM11 epitope were located in the wall of the basal region of the swollen embryo, scale bar = 50 μm; after 3 and 4 weeks of culture, in the enlarged embryo with the rupture of the seed coat, scale bar = 50 μm **(C)**, and the developing protocorm, scale bar = 100 μm **(D)**, the signals of JIM 11 epitope only remained in the surface of the basal protocorms.

TABLE S1 The primers used in the quantitative PCR analyses.
