# Supplementary figures and images for: Immunolocalization and Changes of Hydroxyproline-Rich Glycoproteins During Symbiotic Germination of Dendrobium officinale
Source: Front Plant Sci. 2018 Apr 25;9:552. doi: 10.3389/fpls.2018.00552 (PMC5996918; doi:10.3389/fpls.2018.00552)

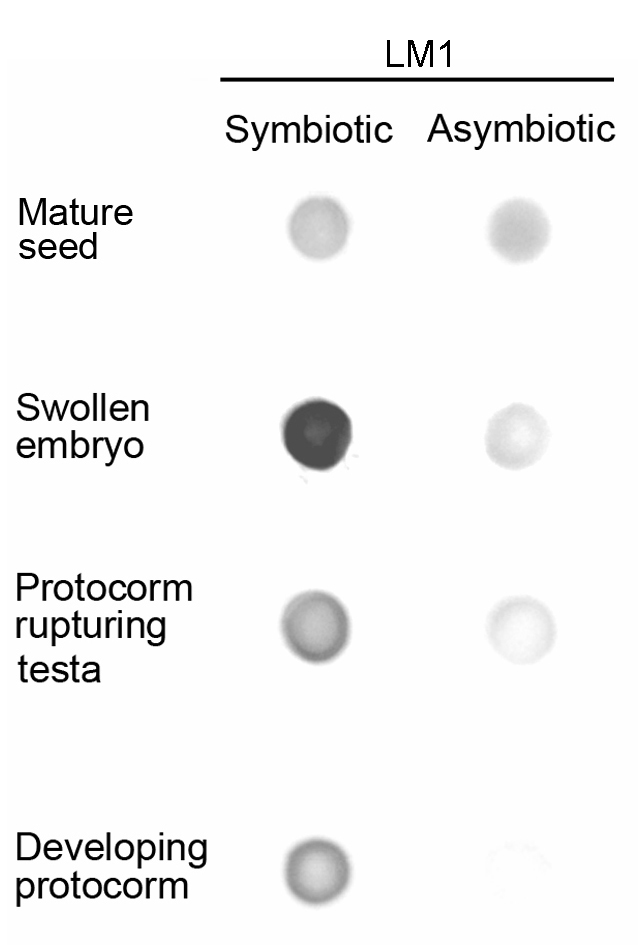

Supplement: FIGURE S1 — Semi-quantitative analysis by immunodot blots of the relative abundance of LM1 epitope in extracts prepared from the mature seeds and developing protocorms in the symbiotic and asymbiotic cultures. [file Image_1.JPEG]

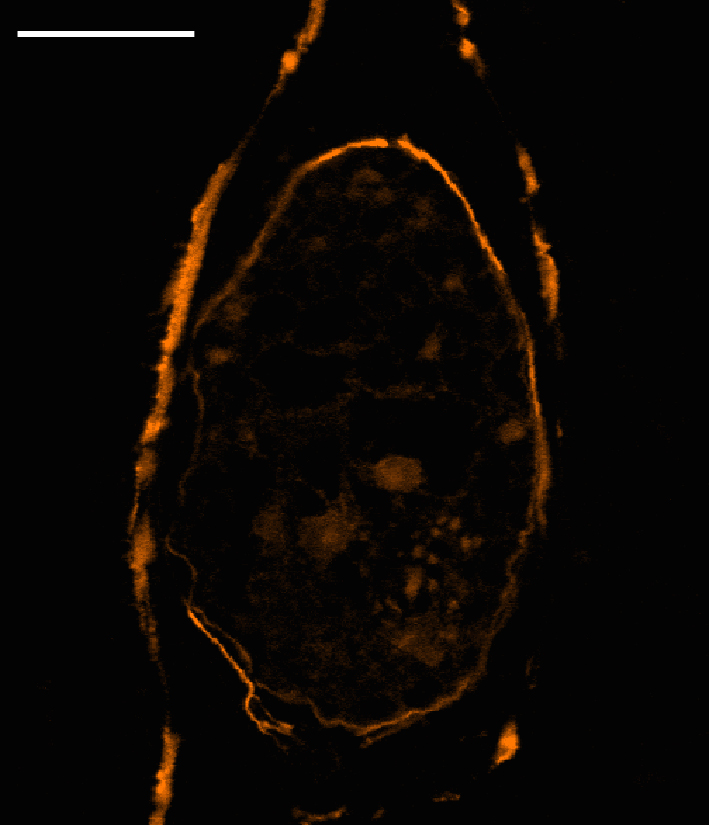

Supplement: FIGURE S2 — In the negative control of immunofluorescence localization of JIM11 epitope, no signal can be detected in the enlarged embryo after incubation without the primary antibody of JIM11. Scale bar = 50 μm. [file Image_2.JPEG]

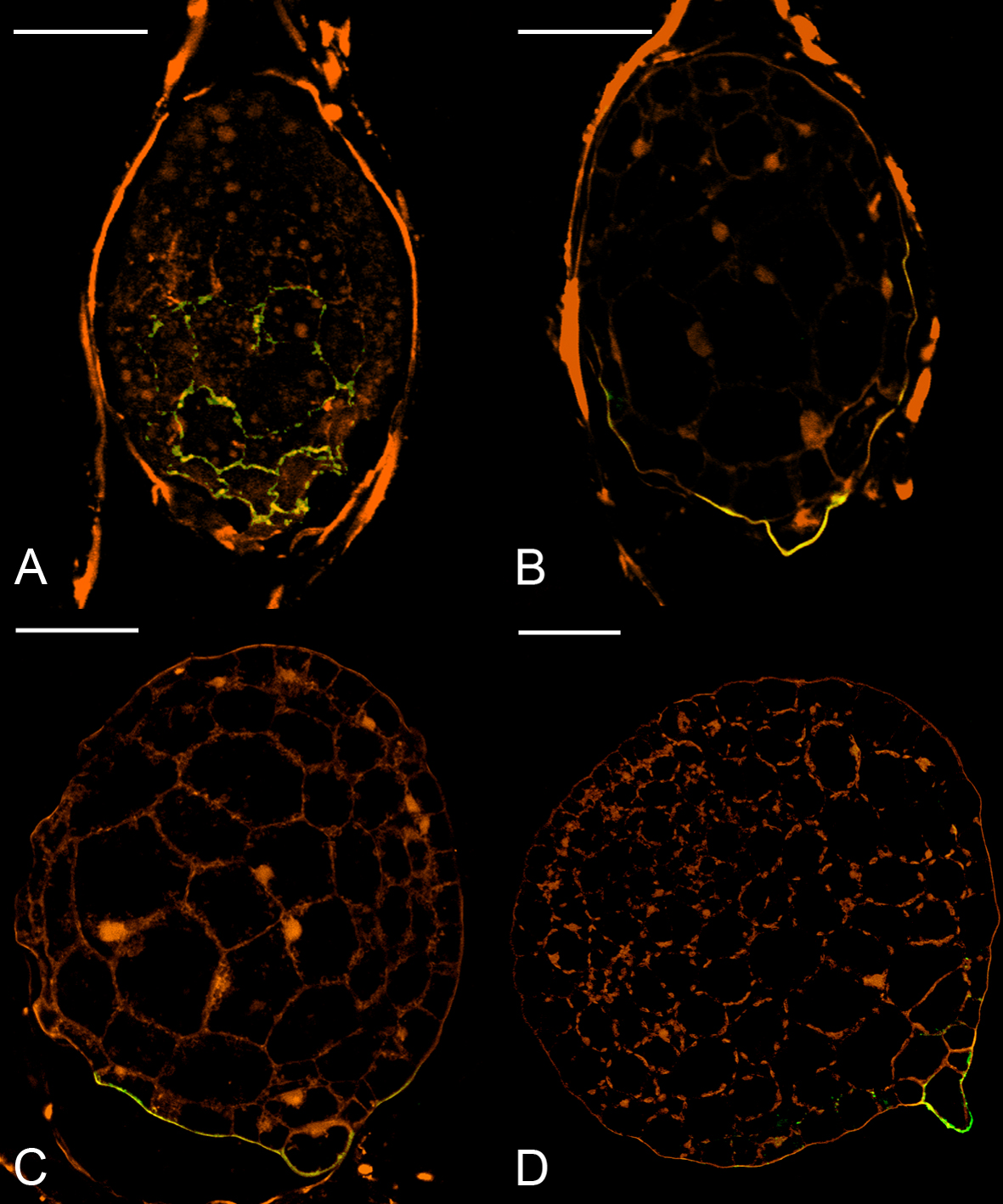

Supplement: FIGURE S3 — In the asymbiotic germination culture, (A) after 3 days of culture, the signals of JIM11 epitope (green color) were located in the walls of the middle and basal regions of the imbibed embryo, scale bar = 50 μm; (B) after 2 weeks of culture, a few signals of JIM11 epitope were located in the wall of the basal region of the swollen embryo, scale bar = 50 μm; after 3 and 4 weeks of culture, in the enlarged embryo with the rupture of the seed coat, scale bar = 50 μm (C), and the developing protocorm, scale bar = 100 μm (D), the signals of JIM 11 epitope only remained in the surface of the basal protocorms. [file Image_3.JPEG]
