# Supplementary material for: Immunolocalization and Changes of Hydroxyproline-Rich Glycoproteins During Symbiotic Germination of Dendrobium officinale
Source: Front Plant Sci. 2018 Apr 25;9:552. doi: 10.3389/fpls.2018.00552 (PMC5996918; doi:10.3389/fpls.2018.00552)
Supplement: TABLE S1 — The primers used in the quantitative PCR analyses. [file Table_1.DOCX]

Table S1. The primers used in the quantitative PCR analyses.

| Unigene ID | Forward primer (5-3') | Reverse primer (5-3') |
| --- | --- | --- |
| actin | TTAGCTGGTCGTGACCTGACTGAT | AACGGAACCTCTCAGCTCCAATT |
| CL493.Contig2 | AATCACCACCACCGCCATCAC | TGGAGGAGGTGGAGAGGGAGAT |
| CL9853.Contig1 | CCATCAGCATCACCGCCATCAA | TCTCCAGCCTCACAAGTCACCT |
| Unigene4313 | TTCTTCCTCCCGTCAAGAAC | AGAGCTAAAGCCATGGTTGG |
| Unigene5337 | CTATATCCGCCACCGCCAACT | GGCTAGGATCAGAAGGCAGAGG |
